# Hand hygiene, knowledge and training motivational drives: findings from a survey in a university hospital

**DOI:** 10.3389/fpubh.2024.1421324

**Published:** 2024-12-18

**Authors:** Maria Incoronata Corbo, Malgorzata Wachocka, Maddalena Pozzi, Marco Cioce, Michele Di Donato, Matteo Raponi, Domenico Pascucci, Eleonora Marziali, Massimo Fantoni, Rita Murri, Sara Vincenti, Carmen Nuzzo, Giuseppe Vetrugno, Andrea Cambieri, Patrizia Laurenti

**Affiliations:** ^1^Health Management, Fondazione Policlinico Universitario A. Gemelli IRCCS, Rome, Italy; ^2^Section of Hygiene, Department of Life Sciences and Public Health, Università Cattolica del Sacro Cuore, Rome, Italy; ^3^Direction of Health Professionals, Fondazione Policlinico Universitario A. Gemelli IRCCS, Rome, Italy; ^4^Unit of Hospital Hygiene, Department of Woman and Child Health and Public Health, Fondazione Policlinico Universitario A. Gemelli IRCCS, Rome, Italy; ^5^Department of Safety and Bioethics, Università Cattolica del Sacro Cuore, Rome, Italy; ^6^Unit of Infection Disease, Department of Medical and Surgery Sciences, Fondazione Policlinico Universitario A. Gemelli IRCCS, Rome, Italy

**Keywords:** hand hygiene, healthcare-associated infections (HCAIs), alcohol hand rub, compliance, survey

## Abstract

This study assessed healthcare workers’ (HCWs) knowledge and adherence to hand hygiene principles in a large Italian university hospital, focusing on identifying knowledge gaps and evaluating training effectiveness. A specifically designed 17-item questionnaire, based on the World Health Organization (WHO) Hand Hygiene Knowledge Questionnaire, was used to measure theoretical knowledge, the role of training, and the impact of experience. The survey had an 8% participation rate (542 responses from a total of 6,749 HCWs), with higher responses among nurses and doctors. Results showed moderate knowledge of hand hygiene protocols, averaging 74%, but revealed gaps in comprehension, particularly in the correct use of hand rub and post-contact sanitation. Logistic regression analysis identified significant predictors of knowledge and adherence, with professional characteristics such as professional qualifications and length of service influencing outcomes (*p* < 0.05). The findings confirm the strong correlation between hand hygiene knowledge and compliance in reducing healthcare-associated infections (HAIs). Continuous education and customized interventions, including targeted training and feedback, are essential for targeting weak points and improving adherence. These insights emphasize the importance of ongoing training and monitoring to enhance hand hygiene practices, promote a culture of patient safety, and, as a consequence, reduce the incidence of HAIs.

## Introduction

Hands of healthcare workers (HCWs) are known to be the main transmission carrier of microbial agents, contributing to their spread (1, 2). Thus, hand hygiene and compliance with the recommendations regarding infection prevention help to reduce the risk of transmitting germs. When good hand hygiene is combined with other preventive measures, the World Health Organization (WHO) estimates a possible reduction of Healthcare Associated Infections (HAIs) of up to 70% (3).

HAIs are the most frequent and serious complications of health care (4), with a significant clinical and economic impact. The European Centre for Disease Prevention and Control point prevalence survey 2022–2023, reported that the prevalence of patients with at least one HAI in the European Union/European Economic Area sample, was 7.1% (country range: 3.1–13.8%) (5).

Hand hygiene has long been considered a cornerstone in risk prevention, and the WHO indicates 80% hand hygiene compliance as the safe level for a lower incidence of HAIs. The single most effective measure for the prevention of infections in different care settings is hand hygiene, with the alcoholic solution considered the gold standard when hands are not visibly soiled (6–8). Alcohol-based hand rub consists of rubbing the antiseptic agent that reduces or inhibits the growth of microorganisms over the entire surface of the hand, without either the need for an exogenous source of water, or for rinsing or drying with towels or other devices (9).

The correlation between hand hygiene among healthcare personnel and the reduction of HAIs is greater if its basic principles are always respected. These must be known by the staff and applied consequently. According to the WHO recommendations expressed in ‘WHO Guidelines on hand hygiene in health care 2009’ (9), the basic principles of good hand hygiene can be identified in the following: hand hygiene is practiced in the 5 Moments identified by the WHO (before touching a patient, before cleaning/aseptic procedure, after body fluid exposure risk, after touching a patient, after touching patient surroundings); the right technique and timing are complied with; the choice between rubbing and washing meet the correct criteria; gloves are used according to proper indications and hand hygiene is carried out before putting them on and after removing them; the hands, during service, are without jewelry and watches. Application of these standards requires HCWs to have theoretical knowledge, strong motivations, and periodic updates.

Several studies, including recent ones, have investigated staff knowledge on hand hygiene in relation to its impact on compliance results, including finding organizational and individual factors that might implement good practice (10–18). Most surveys (18–27) report levels of knowledge and compliance with hand hygiene, which are often correlated with each other, in a range from 60 to 90%. Research carried out at a large Greek university hospital showed a poor staff knowledge level (an average of 54.3%) but a satisfactory compliance (74.03%); the authors attributed this last outcome to routine post-pandemic habits more than education and documented practice; still, despite the apparent conflict in the results, they emphasized the importance of specific recurring training in healthcare facilities (28). In support of this, they referred to the numerous publications in the field (19–27, 29–32) from which some common elements emerge, albeit the heterogeneity of contexts, such as: a better knowledge and compliance in nursing staff compared to doctors, a better performance by those who have recently attended a course on infection risk prevention, and the favorable role of experience on adherence to guidelines.

The assessment of health professional knowledge on hand hygiene principles, therefore, appears to be fundamental to understanding which factors can influence compliance, so that it may help plan actions that promote practice (15–18).

In a review of 41 studies (33) describing frequently used improvement strategies and the related determinants of behavior change leading to good adherence to hand hygiene programs, it emerges that knowledge is a decisive factor, if associated with awareness for one’s actions.

In this context, where the specific organizational culture of the staff plays a pivotal role, the primary objective is to assess the level of knowledge among healthcare and cleaning personnel by targeting a representative sample of at least 10% of eligible individuals. The data collected will serve as a foundation for designing and implementing future training interventions aimed at enhancing the knowledge base of healthcare workers, ultimately optimizing hand hygiene practices and promoting better infection control. Secondary objectives were the evaluation of the effectiveness of the training recently offered, and the role of experience and length of service (understanding whether older professionals can act as role models or if, vice versa, they should be better accompanied and updated).

## Materials and methods

### Context and participants

Potentially eligible recipients of this survey are healthcare professionals (all categories, including medical residents) working at the Fondazione Policlinico Universitario “Agostino Gemelli” IRCCS, a highly complex university hospital in Rome with 1,581 beds. At the time of the survey, the staff consisted of 6,749 health workers distributed as follows: 1,156 medical staff, 2,130 medical resident staff, 2,280 nursing staff, 705 auxiliary staff, 478 technicians and other health professions. The expected target was the achievement of at least 10% of all operators. During the period in which the survey was carried out, a specific short all-user course on hand hygiene had already been made available on the company training platform and recommended to all target users. In addition to this training, and the formal audit program on the topic, a hand hygiene promotion program based on the WHO multimodal strategy, with the application of multiple targeted interventions of various nature, had already been in place for about a decade, including the development of hand hygiene policies, extended performance monitoring with internal training of observers and data return, hand dress code campaigns, and the posting of dedicated posters and reminders.

### Study design

A specially constructed questionnaire inspired by the WHO survey form (WHO Hand Hygiene Knowledge Questionnaire for Health-Care Workers-revised 2009-) was used. Some minimal changes from the original tool were necessary to adapt it to the organizational and cultural context of the facility (Appendix 1). The modifications made to the questionnaire were processed and approved by the Committee for Good Hand Hygiene Practice, a hospital commission of experts with permanent activity on the topic, including training and education, made up of doctors, nurses and prevention technicians operating in the field of hygiene and risk management. The questionnaire, made up of a total of 17 items, includes a first section which collects socio-demographic data (9 questions), a question on staff training, one on the habit of using the alcoholic solution, and 12 questions which probed the theoretical knowledge on the subject. The latter were all multiple-choice questions with 4 proposed options, sometimes with only a single possibility of correct answer, others with multiple possible answers. In this case, an answer is considered correct when it contains at least one correct option and no wrong option (any incorrect answer). Participant selection was performed using random sampling until the expected target was achieved. Participation in the survey was on a voluntary basis. A communication/information e-mail was sent to the heads of Departments and to the coordinators of the health professions with the request that it be released to all personnel of their staff, to inform them of the imminent survey, and in order to obtain collaboration and support from the head chiefs to encourage broad participation. Participants were provided with a link to complete the questionnaire in April 2023, and a reminder was sent to encourage participation. Participants were guaranteed anonymity, if desired, but also the possibility of declaring their identity. It was also assured that the results would only be presented in aggregated form. The data collection and processing were carried out through a Microsoft Form platform by the health professionals in force at the Hospital Hygiene Unit and by Health Profession Department, Risk Management, Quality and Safety Manager, and jointly evaluated by the Hand Hygiene Good Practice Committee of the hospital.

### Statistical analysis

The sample was described in its socio-demographic characteristics using the main techniques of descriptive statistics. In particular, the qualitative variables were reported as absolute and relative frequencies (percentages), while the quantitative type variables were summarized, with the mean and standard deviation and/or with the median and interquartile range if the assumption of normality was not respected in the distribution of variables. To verify “normality,” the scores obtained with a study of the mean values, the Standard Deviation (SD) and the distribution using the S-France test were analyzed. Inferential tests were also performed on the dataset stratified by gender, age group, professional qualification, and length of service. In particular, the chi-square test was used for qualitative variables. Logistic regression was used to find the best fitting model to describe the relationship between variables, adjusting for the effect of the other variables in the model. The results were considered statistically significant with a *p* value <0.05. All analyses were conducted with STATA 17.0 BE—Basic Edition software.

## Results

Among 6,749 health workers questionnaire recipients, 542 (8% of the total) completed the questionnaire. The average age of the potentially eligible was 45 years old, 62% of which were female.

Table 1 shows the characteristics of the sample. The median age was 35 years (IQR: 27; 50) and about three-quarters were female (72%, *n* = 388).

**Table 1:** Characteristics of the sample.

**Age (years)**		
Median (IQR)	35 (27; 50)	
**Gender**	**N**	**%**
Male	154	28.4
Female	388	71.6
**Professional title**	**N**	**%**
Medical staff	94	17.4
Medical resident staff	121	22.4
Nursing staff	206	37.9
Biologist/pharmacist staff	22	4.1
Rehabilitation therapist	14	2.6
Healthcare assistants	10	1.8
Technician	12	2.2
Domestic and cleaning staff	6	1.1
Other health professions	57	10.5
**Length of service**	**N**	**%**
1–5	170	37.6
6–10	44	9.7
11–15	34	7.5
16–20	39	8.6
21–25	40	8.8
26–30	20	4.4
31–35	39	8.6
>35	66	14.6
Missing	90	–

Most of the sample (87.2%, *n* = 472) reported receiving hand hygiene training in the last 3 years. Almost everyone (97%, *n* = 525) usually uses an alcohol-based product for hand hygiene (Table 2).

**Table 2:** Percentage of correct answers of the questionnaire.

Questionnaire	Yes/Correct answer	
	N	%
Have you received hand hygiene training in the last 3 years (including through FAD)?	472	87.2
Do you usually use an alcohol-based product for hand hygiene?	525	97
Q1: Which of the following is the major route of cross-transmission of potentially pathogenic germs between patients in a healthcare facility?	466	85.8
Q2: Which of the following hand hygiene actions prevents the cross-transmission of germs to the patient?	223	41.1
Q3: Which of the following hand hygiene actions prevents the HCW from getting infected?	413	76.2
Q4: What is the minimum time it takes for an alcohol-based product to kill most of the germs on your hands?	349	64.3
Q5: After contact with the patient, for which of the following infections should you wash your hands with soap and water and not with the alcohol-based product?	371	68.3
Q6: Rubbing with an alcoholic solution is quicker than washing your hands: true or false?	401	73.8
Q7: Hand rubbing causes dry skin more than hand washing: true or false?	412	75.9
Q8: Hand rubbing is more effective against germs than hand washing: true or false?	165	30.4
Q9: It is recommended to wash and rub your hands in sequence: true or false?	392	72.2
Q10: Before putting on gloves, hand hygiene is necessary: true or false?	438	80.7
Q11: If you do not touch the patient, but only the surfaces that surround him (his bed, bedside table, IV drip.), it is not important to sanitize your hands before leaving: true or false?	524	96.5
Q12: If you sanitize your hands correctly, the hygiene of the rings worn, and the safety of the hand is also guaranteed: true or false?	504	92.8

Among the 12 questions designed to evaluate knowledge of hand hygiene, the mean percentage of correct answers was 74.4%.

With reference to the questionnaire, the highest percentage of correct answers was reported for the question “Q11: If you do not touch the patient, but only the surfaces that surround him (his bed, bedside table, IV drip), it is not important to sanitize your hands before leaving: true or false?” while the lowest percentage was reported for the question “Q8: Hand rubbing is more effective against germs than hand washing: true or false?” (Table 2).

Table 3 shows that females had a greater knowledge about Q4 and Q7 (67.5% of correct answers vs. 56.5%, *p* = 0.016 and 78.6% of correct answers vs. 68.8%, *p* = 0.016).

**Table 3:** Percentage of correct answers by characteristics of the sample and results of the univariate analysis.

	Q1	*p*-value	Q2	*p*-value	Q3	*p*-value	Q4	*p*-value	Q5	*p*-value	Q6	*p*-value
Gender												
M	88.3	0.324	37.7	0.299	77.9	0.553	56.5	**0.016**	68.2	0.932	77.3	0.272
F	85		42.5		75.5		67.5		68.6		72.7	
Professional title												
Medical staff	91.5	**<0.001**	41.5	**0.001**	79.8	**0.029**	55.3	0.122	66	**<0.001**	75.5	0.568
Medical resident staff	87.6		43.8		80.2		56.2		68.6		78.5	
Nursing staff	92.2		32.7		77.6		71.2		83.9		74.6	
Biologist/pharmacist staff	68.2		77.3		77.3		68.2		31.8		63.6	
Rehabilitation therapist	85.7		21.4		92.9		71.4		64.3		85.7	
Healthcare assistants	70		70		50		70		80		70	
Technician	58.33		50		58.3		58.3		50		66.7	
Domestic and cleaning staff	100		50		66.7		66.7		66.7		66.7	
Other staff	66.7		49.1		61.4		70.2		35.1		64.9	
Staff that received hand hygiene training in the last 3 years												
Yes	86.2	0.871	40.3	0.233	76.3	0.869	65.5	0.224	72	**<0.001**	76.1	**0.007**
No	85.5		47.8		75.4		58		44.9		60.9	
Age												
21–30	83.8	0.463	40.2	0.441	76.5	0.129	64.7	0.106	71.1	0.226	76.5	0.407
31–40	83.8		41		75.2		57.3		61.5		76.9	
41–50	87.5		45		76.2		58.7		63.7		67.5	
51–60	90.6		36.8		73.6		72.6		72.6		69.8	
>61	89.7		55.2		96.5		72.4		75.9		76.7	
Length of Service												
1–5	84.7	0.217	38.2	0.473	80	0.852	60.6	0.052	68.2	0.708	78.8	0.335
6–10	79.5		52.3		75		61.4		65.9		63.6	
11–15	94.1		47		73.5		55.9		67.6		76.5	
16–20	84.6		35.9		74.4		66.7		66.7		74.4	
21–25	92.5		42.5		75		70		72.5		75	
26–30	95		35		85		65		85		65	
31–35	92.3		35.9		71.8		71.8		76.9		64.1	
>35	80.3		50		72.7		83.3		65.1		77.6	

	Q7	*p*-value	Q8	*p*-value	Q9	*p*-value	Q10	*p*-value	Q11	*p*-value	Q12	*p*-value
Gender												
M	68.8	**0.016**	31.8	0.661	74	0.537	78.6	0.404	94.2	0.062	92.9	0.940
F	78.6		29.9		71.4		81.7		97.4		93	
Professional title												
Medical staff	75.5	0.414	34	0.451	72.3	**0.001**	80.6	0.499	96.8	0.959	91.5	**0.001**
Medical resident staff	67.8		24		69.4		76.9		96.7		90.1	
Nursing staff	78.5		30.2		79.5		84.4		96.6		98.5	
Biologist/pharmacist staff	68.2		31.8		31.8		72.7		95.5		90.9	
Rehabilitation therapist	85.7		42.9		78.6		92.9		100		92.9	
Healthcare assistants	80		10		70		80		100		100	
Technician	75		41.7		66.7		75		91.7		75	
Domestic and cleaning staff	83.3		33.3		83.3		100		100		100	
Other staff	82.5		36.8		64.9		77.2		94.7		84.2	
Staff that received hand hygiene training in the last 3 years												
Yes	76.1	0.697	31.1	0.394	74.4	**0.002**	81.6	0.347	96.8	0.270	93	0.938
No	73.9		26.1		56.5		76.8		94.2		92.8	
Age												
21–30	69.6	**0.026**	25	**0.024**	70.1	0.795	82.4	0.737	96.6	0.691	92.2	**0.040**
31–40	72.7		24.8		72.7		78.6		94.9		87.2	
41–50	81.3		37.5		76.3		82.5		97.5		96.3	
51–60	83		34.9		69.8		79.3		96.2		97.2	
>61	86.7		46.7		76.7		73.3		100		93.3	
Length of service												
1–5	68.8	**0.044**	25.3	**<0.001**	69.4	0.142	82.9	0.982	97.7	0.872	91.2	0.430
6–10	70.5		15.9		70.5		79.6		97.7		93.2	
11–15	85.3		47.1		79.4		82.4		94.1		97.1	
16–20	82.1		38.5		74.4		82.1		97.4		94.9	
21–25	75		22.5		77.5		80		95		100	
26–30	80		35		95		85		100		100	
31–35	89.7		28.2		66.7		84.6		94.9		94.9	
>35	83.6		52.2		82.1		77.6		95.5		92.5	

Bold values represent the statistically significant results at the *p* < 0.05 level.

Furthermore there was a significant association between the professional title and greater knowledge about many questions of the questionnaire (medical staff and medical resident staff record the most correct answers for the questions Q1 and Q3, nursing staff for the questions Q1, Q5, Q9 and Q12, biologist/pharmacist staff for Q2, rehabilitation therapists for Q3 and Q9, healthcare assistants for Q2, Q5 and Q12 and Domestic and cleaning staff for Q1, Q9 and Q12).

Staff that received hand hygiene training in the last three years record a greater knowledge about Q5, Q6 and Q9 (72% of correct answers vs. 45%, *p* < 0.001, 76% of correct answers vs. 61%, *p* = 0.007 and 74% of correct answers vs. 57%, *p* = 0.002).

With reference to Q7, Q8 and Q12 the percentage of correct answers increases with the increase in age (*p* = 0.026, *p* = 0.024, *p* = 0.040).

Personnel with a length of service equal to 11–15 and > 35 years record a greater knowledge about Q7 and Q8 (*p* = 0.044, *p* < 0.001); staff with 31–35 years of service record the greatest knowledge about Q7.

The multivariable linear regression analysis (Table 4) confirmed the association between being a woman and greater knowledge about Q7 (OR = 1.8; *p* = 0.026), staff that received hand hygiene training in the last 3 years and a greater knowledge about Q5 (OR = 0.33; *p* = 0.002). In addition, with reference to Q8 it confirmed that the percentage of correct answers increases with the increase in age.

**Table 4:** Logistic regression model for correct answers and characteristics of the respondents.

		Q1		Q2		Q3		Q4		Q5		Q6	
		OR	*p* value	OR	*p* value	OR	*p* value	OR	*p* value	OR	*p* value	OR	*p* value
Gender	Male	1(reference)		1(reference)		1(reference)		1(reference)		1(reference)		1(reference)	
	Female	0.78	0.488	1.33	0.244	0.71	0.247	1.55	0.065	0.69	0.180	0.90	0.695
Professional title	Medical staff	1(reference)		1(reference)		1(reference)		1(reference)		1(reference)		1(reference)	
	Medical resident staff	0.68	0.518	1.47	0.331	1.17	0.746	1.03	0.93	0.85	0.711	0.87	0.766
	Nursing staff	1.18	0.74	0.63	0.122	1.11	0.769	1.46	0.213	2.69	**0.004**	0.65	0.203
	Biologist/pharmacist staff	0.44	0.303	6.60	**0.008**	1.26	0.757	2.10	0.258	0.46	0.221	0.98	0.973
	Rehabilitation therapist	0.47	0.4	0.39	0.185	4.03	0.204	2.18	0.278	0.93	0.912	1.31	0.747
	Healthcare assistants	0.25	0.098	3.70	0.083	0.26	0.058	1.74	0.463	2.43	0.31	0.75	0.704
	Technician	0.15	**0.033**	4.15	0.101	0.49	0.38	1.47	0.62	0.31	0.14	0.26	0.089
	Domestic and cleaning staff	1.00	-	1.58	0.601	0.40	0.336	1.42	0.704	0.84	0.854	0.45	0.395
	Other staff	0.13	**0.001**	1.34	0.555	0.48	0.182	1.19	0.736	0.08	**<0.001**	0.38	0.071
Staff that received hand hygiene training in the last 3 years	Yes	1(reference)		1(reference)		1(reference)		1(reference)		1(reference)		1(reference)	
	No	0.82	0.683	1.16	0.666	0.83	0.616	1.03	0.926	0.33	**0.002**	0.56	0.099
Age	21–30	1(reference)		1(reference)		1(reference)		1(reference)		1(reference)		1(reference)	
	31–40	1.01	0.984	0.78	0.405	0.93	0.848	0.70	0.239	0.37	**0.005**	0.94	0.858
	41–50	1.27	0.695	0.75	0.511	1.17	0.753	0.57	0.2	0.34	**0.028**	0.47	0.106
	51–60	1.22	0.735	0.58	0.229	1.20	0.71	0.89	0.816	0.26	**0.017**	0.76	0.58
	>61	0.62	0.565	1.07	0.91	9.78	**0.044**	0.76	0.688	0.30	0.109	0.80	0.757
Length of service	1–5	1(reference)		1(reference)		1(reference)		1(reference)		1(reference)		1(reference)	
	6–10	0.62	0.366	2.38	**0.032**	0.91	0.83	1.12	0.78	1.00	0.998	0.59	0.21
	11–15	3.17	0.183	1.72	0.256	0.79	0.654	1.01	0.987	2.29	0.129	1.50	0.451
	16–20	0.77	0.695	1.23	0.679	0.70	0.49	1.55	0.361	1.65	0.365	1.34	0.575
	21–25	1.47	0.627	2.46	0.075	0.52	0.251	1.59	0.376	1.62	0.414	1.24	0.692
	26–30	1.97	0.564	1.77	0.376	1.38	0.684	1.02	0.97	3.59	0.123	0.70	0.59
	31–35	1.57	0.587	1.91	0.257	0.46	0.218	1.31	0.648	2.18	0.258	0.60	0.394
	>35	1.85	0.305	2.41	0.078	0.82	0.704	3.07	**0.047**	3.07	0.079	1.89	0.243

		Q7		Q8		Q9		Q10		Q11		Q12	
		OR	*p* value	OR	*p* value	OR	*p* value	OR	*p* value	OR	*p* value	OR	*p* value
Gender	Male	1(reference)		1(reference)		1(reference)		1(reference)		1(reference)		1(reference)	
	Female	1.80	**0.026**	0.99	0.958	0.68	0.169	1.23	0.488	5.57	**0.011**	0.41	0.110
Professional title	Medical staff	1(reference)		1(reference)		1(reference)		1(reference)		1(reference)		1(reference)	
	Medical resident staff	1.16	0.718	0.77	0.556	1.05	0.906	0.47	0.132	1.30	0.822	2.73	0.167
	Nursing staff	1.12	0.747	0.87	0.674	1.60	0.181	1.33	0.455	0.98	0.977	1.37	**0.004**
	Biologist/pharmacist staff	0.71	0.618	1.24	0.734	0.26	**0.036**	0.63	0.503	1	–	2.02	0.561
	Rehabilitation therapist	1.83	0.473	1.96	0.291	1.17	0.832	2.40	0.424	1	–	1	–
	Healthcare assistants	1.11	0.904	0.17	0.115	1.45	0.655	1.03	0.969	1	–	1	–
	Technician	1.90	0.568	1.06	0.942	0.56	0.486	0.65	0.632	0.11	0.127	0.11	**0.029**
	Domestic and cleaning staff	1.27	0.832	0.52	0.482	1.74	0.635	1.00	–	1	–	1	–
	Other staff	1.60	0.438	1.36	0.554	0.30	**0.026**	0.49	0.204	1.25	0.298	0.63	0.574
Hand hygiene training in the last 3 years	Yes	1(reference)		1(reference)		1(reference)		1(reference)		1(reference)		1(reference)	
	No	0.75	0.429	0.56	0.138	0.61	0.174	0.61	0.201	0.29	0.104	2.07	0.374
Age	21–30	1(reference)		1(reference)		1(reference)		1(reference)		1(reference)		1(reference)	
	31–40	1.24	0.509	1.05	0.886	0.96	0.901	0.62	0.209	1.13	0.896	0.74	0.588
	41–50	1.92	0.188	2.59	**0.036**	0.67	0.414	0.63	0.391	5.56	0.234	2.01	0.503
	51–60	2.12	0.161	2.97	**0.021**	0.23	**0.005**	0.37	0.054	1.68	0.13	0.91	0.922
	>61	3.23	0.128	3.22	0.06	0.22	**0.028**	0.38	0.172	1	–	1.06	0.97
Length of service	1–5	1(reference)		1(reference)		1(reference)		1(reference)		1(reference)		1(reference)	
	6–10	0.92	0.837	0.43	0.097	1.17	0.725	0.65	0.409	1.06	0.965	2.36	0.329
	11–15	2.03	0.227	1.75	0.246	3.00	0.057	0.98	0.968	0.29	0.292	1.17	0.08
	16–20	1.42	0.531	0.84	0.727	2.49	0.104	0.92	0.893	0.25	0.331	1.51	0.707
	21–25	0.70	0.535	0.29	**0.024**	3.20	**0.048**	0.73	0.604	0.04	**0.044**	1	–
	26–30	0.88	0.865	0.63	0.474	2.48	**0.005**	1.23	0.797	1	–	1	–
	31–35	1.79	0.436	0.39	0.109	2.93	0.082	1.30	0.704	0.01	**0.036**	1.30	0.835
	>35	0.96	0.943	1.46	0.451	6.09	**0.002**	0.82	0.736	0.01	**0.049**	2.71	0.236

Bold values represent the statistically significant results at the *p* < 0.05 level.

## Discussion

The survey presented provided a useful and interesting framework to the promoting group. In relation to the objectives of the survey and before discussing the results, it is necessary to report some reflections on the level of participation of the recipients, that is to say all the staff of the university hospital, for a total of 6,749 operators.

### Survey participation

The internal participation percentage was 8%, against the expected minimum of 10%.

Participation was greater for the medical and nursing categories (17.4% for structured doctors, 22.4% for specialists and 37.9% for nurses), considered the categories of greatest interest for the survey. Regrettably, a poor representativeness of healthcare assistants was noted (1.8% of the sample, equal to approximately 10 healthcare assistants out of 555), whose operators, due to the type of their work activity, are extremely involved in this hand hygiene good practice as they are engaged in direct assistance. Nonetheless, the different participation rate of the professional categories could have been influenced by the greater propensity and sensitivity of doctors and nurses to periodic checks and their habit of measuring their own performance. The limited participation of healthcare assistants could thus be correlated to a lower attitude toward self-monitoring, a characteristic that should be encouraged in any case, for a professional figure which, although introduced into our reality quite recently, is establishing itself in an increasingly positive way.

The median age of the participants was 35 years, and over 71% were female operators. The latter data can partly be explained by the greater female presence in the facility, but it exceeds, in proportion, the female percentage in the hospital (62%).

Most of them had received specific training in the last 3 years (87.2%), a sign of the structure’s ability, although vast, articulated and complex, to systematically take care of the training and updating of staff.

The extremely widespread habitual use of the alcohol-based solution for hand hygiene (97% of participants) confirms that the promotion on the use of the product carried out by the structure through training and information campaigns was very effective. Alcoholic rub is the method preferred by operators and in line with the WHO recommendation which indicates it as the gold standard for hand hygiene.

The conducted survey, together with the results obtained, allowed, albeit partially, to acquire information on the two objective domains of the investigation: the average preparation of all operators and within the different professional categories, and the identification of weak points in knowledge; and on the two sub-objectives concerning the evaluation of the effectiveness of the training and the role of experience and length of service, so that it will be possible to offer, in the near future, an increasingly functional education aimed at the skills of each individual.

### Survey objectives and reflections on the results

- 1st domain, the average preparation of the operators. This was, for the 12 cognitive questions, equal to 74.4%, a value that we consider rather satisfactory considering the complexity of the structure and the difficulty in making learning widespread and pervasive. The data is particularly eloquent when compared with the result achieved exactly 10 years earlier in the same structure (34), in a period in which the multimodal strategy for the promotion of hand hygiene was not yet applied. Although acquired with a different survey tool and methodology, an average level of knowledge among staff emerged of 37.8% ranging from 25.6% for domestic and cleaning staff to 50% for doctors. The survey carried out at the time consisted of only three questions, included in a questionnaire consisting mostly of items on antisepsis and disinfection topics and included a sample of 150 operators (49.3% nursing staff, 17.3% domestic and cleaning staff, 16% doctors and 17.3% medical resident staff; the figure of healthcare assistant was not yet present). The engagement made in this decade by the infection prevention and control group and by the health management of the facility also manifests itself in this increase in knowledge and awareness, the ultimate benefit of which is the increase over the years in compliance with the hand hygiene in operational units, effectively observed through continuous monitoring: from 34.4% in 2016 (unpublished data resulting from internal hospital survey) to 81.4% in 2022, with a constantly rising trend.

Nevertheless, there are specific knowledge gaps still to be filled, as demonstrated by the more in-depth analysis of the responses, illustrated below in the second domain.

The knowledge level seems to depend, for 2 questions out of 12, on gender (females perform best), for 4 questions out of 12 on qualification (nurses perform best), and for 3 questions on age and length of service, with the highest groups - people with greater age, and with 11–15 years of service or > 35 years of service – performing better. Work experience is undoubtedly an asset for the sedimentation of knowledge and for being acted upon appropriately; furthermore, work continuity exposes and, at the same time, encourages more frequent training ‘refreshers’.

Furthermore, the training received makes a difference, even if for only 3 questions out of 12: this result does not seem, at first glance, to explicitly highlight the positive role of training, although its indirect effectiveness is intuited. In fact, where training is carried out on a constant basis, a widespread underlying culture is also created through ‘contagion’ among workers, who, despite not having attended formal training sessions, can benefit from the knowledge transmitted to them by peers or trained superiors.

Other studies from industrialized countries report different percentages of knowledge among health workers. Sinopidis et al. (35) report an acceptable level of knowledge of 54.3% in personnel achieved by formal training in the last 3 years. Hammerschmidt and Manser (10) highlight that only one nurse in five has correct knowledge of the practical implementation of the concepts learned in the training.

- 2nd domain, theoretical concepts and specific knowledge. Surprisingly, the highest percentage of correct answers was reported for question 11 “If you do not touch the patient, but only the surfaces that surround him (the bed, the bedside table, the IV drip), it is not important to sanitize the hands before going out: true or false?.” This result does not seem consistent with the observations conducted in the field within our hospital, which instead identify, in the 5th Moment of hand hygiene (‘after contact with the patient’s surroundings’), the generally weakest element. It is known, however, that knowledge does not always have a direct impact on practice (36, 37). Furthermore, one cannot exclude the possible induction which the very presence of the question in the survey may have had on the person filling out the questionnaire: this could have pushed the operator to consider the surrounding environment as important (as it is) in the transmission of germs and led them to the correct answer. From this perspective, the question may have added useful information from a learning point of view, increasing the knowledge of those who responded to the interview.

The question with the lowest percentage of correct answers was question 8 “Is hand rubbing more effective against germs than hand washing: true or false?.” (Table 2). Here, a greater knowledge on the effectiveness of the alcohol-based product was expected. Fortunately, since the alcoholic solution is very widespread in the structure, internal monitoring data reveal that it is, in practice, the most used hygiene method. This is confirmed by the large audience that regularly uses it (97% according to this survey). Older operators seem to be more knowledgeable on this point.

The time necessary for a correct surgical friction (question 4) is known in 64.3% of the interviewed HCWs. This data may have been influenced by the temporary introduction, during the Covid-19 pandemic, of an alcoholic product with a different application time required (1 min versus 30 s of proven effectiveness of 70% alcohol) thus generating confusion in professionals and workers. In the investigation by Aiello et al. (12), again relating to nursing homes, only 40% of the nurses knew the correct application time of the alcoholic solution, while in the research by Hammerschmidt and Manser (10) this percentage rises to 79%.

Regarding question 5 (soap and water as the recommended method for exposure to *Clostridioides difficile*), the general lack of knowledge is worrying, but it must be kept in mind that the group of participants also includes qualifications not involved in direct care (biologists/pharmacists, other staff). However, the good knowledge on this point among nurses and healthcare assistants, those who mostly work in contact with the patient and are called upon to contain the risk of spreading germs, is comforting. Another encouraging element is the effectiveness of the training on this topic, with a significant correlation between those who received training and the correctness of the response. This urges us to continue along this path with conviction. The topic, however, needs to be refreshed during the next training sessions, to better transmit it.

Some elements can be deduced from the significant association between the professional title and greater knowledge for many questions. The first, already reported, is the greater (albeit modest) general knowledge among nurses: the correlation between being a nurse and the correct answers emerges for 4 questions out of 12, while all the other categories show a correlation with the correct answers for a number of questions from 0 to 3. The second, some of the theoretical concepts of transmission of microorganisms and infection, in a variable manner, seem to be consciously acquired by physicians, medical residents, nurses, domestic and cleaning staff (knowledge of the main transmission route), healthcare assistants, biologists and pharmacists (prevention of cross-transmission). However, much remains to be done, with the necessary conceptual insights: knowing the motivation for action greatly supports good practice. The third element has also already been stated and commented on: the correct method of hand hygiene in case of *Clostridioides difficile* (soap and water) is better known among nurses and healthcare assistants Finally, nurses, healthcare assistants and domestic and cleaning staff appear more aware, compared to other categories, of the fact that the jewelry they wear is not adequately sanitized when they sanitize their hands, so it is a good rule not to wear it at all. The facility is investing heavily in the ‘No jewelry in hospital’ campaign, with an ambitious work in progress tending toward a bare hand, and wrists free of jewelry, wristwatches and other items.

- The two sub-objectives: evaluate the effectiveness of the training, and the role of age and length of service as factors. The correlation between training received and level of knowledge has already been mentioned. This correlation, if seen in general, appears to be lower than expected, but, when seen in detail of the individual items on the explored topics, it emerges that the training proves to be influential for a particularly critical content, such as that on the correct method of hand hygiene in the case of *Clostridioides difficile*. The training also affects awareness concerning the fact that handwashing takes longer to be effective than hand rubbing and understanding that rubbing immediately after washing with soap and water is not recommended.

The apparent limited influence of training (for only 3 out of 12 answers is there a correlation with the correct answers) associated however with the above-average level of knowledge of the operators (over 74%), can be interpreted as a positive trace of widespread knowledge, which permeates the facility regardless of the time interval between the training interventions received. This may be the case of HCWs who had received specific training over the previous 3 years, who have retained the knowledge or who acquire it and pass it on to new recruits within their operational units. This knowledge, strengthened by example, is also transmitted between peers and can grow with experience and length of service also through personal reading and participation in broader conferences. In any case, we are called, as trainers, to reach everyone with greater frequency and constant periodicity, insisting on those important themes necessary to guide the gesture (such as hand hygiene) and for which greater weakness emerges.

Regarding the length of service of the workers in the facility, what emerged was not so obvious, therefore it could be considered an added value for the structure and not an area of weakness. The expertise of workers with more years of activity can make them effective role models: according to Merton’s definition, people who offer a positive example worthy of imitation (14). This represents a precious resource for the entire community.

### Implications for practice

Hand hygiene, for some time considered a cornerstone in the prevention of hospital infections, reached a peak in March 2020 in the field of research and practice. Most of the studies reported a compliance rate between 60 and 90% (19–27). Despite all the resources involved, also for the facility involved in this investigation, although compliance has reached the levels recommended by the WHO, at least 80%, it is not always possible to keep it constant and of high quality. To date, direct observation performed by a trained observer represents the reference standard for assessing the degree of adherence to hand hygiene, through timely collection and accurate evaluation. However, there are many limitations related to this methodology including the Hawthorne effect (greater adherence by operators in response to their awareness of being observed), the need for dedicated staff, control acceptance, costs and time required. The discrepancy between adherence to hand hygiene perceived by HCWs, systematically overestimated, and that which is observed and measured, pushes us to seek new approaches to awareness of a safe hand, also moving in the direction of behavioral sciences, behavioral insights - studies on behavior and how to influence it (35, 36, 38, 39). These help to understand human behavior and underlying decision-making processes. Alongside traditional cognitive tools, this knowledge is fundamental for designing innovative practical solutions to promote virtuous behaviors such as hand hygiene at the right time. Nonetheless, theoretical preparation and cognitive background remain fundamental for optimizing practice. Hammerschmidt and Manser (10) report that individual factors such as knowledge of the 5 Moments, behaviors including not wearing jewelry on the hands and wrists, and the application of learning from the most recent trainings, are important prerequisites for the prevention of infections. Yeung et al. have shown how hygiene programs and training in care homes can effectively increase adherence to alcohol rub and reduce the incidence of major infections (14).

The results of the survey offer us concrete indications for planning targeted educational interventions, identifying the professional categories that need specific attention, and the concepts to strengthen or deepen.

The investigation has some limitations. Firstly, the participation rate, which did not reach the desired level, thus providing a small sample of the working reality. The internal participation percentage was 8%, against the expected minimum of 10%. The desired minimum objective, although low, was agreed considering the structural and organizational size of the hospital. A wider inclusion of the sample would have entailed a greater lengthening of time, not compatible with the deadlines of the promoting group. We therefore wanted to favor a method that was streamlined and quick in the collection and subsequent processing of data, including participation on a voluntary basis and in no way mandatory. Mandatory participation, together with widespread and persuasive promotion, would have made it possible to reach many more workers. Those who participated could be among the most motivated or attentive, although we do not have the information to say so.

The results of this study suggest that healthcare facilities can customize educational programs to target gaps in hand hygiene knowledge, especially for professional categories with lower awareness. By organizing more intensive training sessions, interactive workshops, and hands-on exercises, healthcare workers’ retention and application of hand hygiene protocols could be enhanced. The findings also allow for the refinement of hand hygiene protocols targeted to the specific challenges faced by different healthcare roles, which would promote better adherence and reduce healthcare-associated infections (HAIs). Continuous monitoring and feedback, based on identified knowledge gaps, could help to emphasize proper hygiene techniques, while periodic audits and real-time feedback could sustain compliance and enable punctual interventions. The study’s results may also be useful for revising institutional policies on infection control, aligning them with WHO guidelines, and promoting a more rigorous application of hand hygiene standards among worst healthcare workers’ categories. By promoting a stronger culture of patient safety, senior healthcare professionals and managers could use the findings to stimulate a more collaborative environment where hand hygiene practices become a shared responsibility.

The communication then took place in an exclusively computerized manner, without resorting to other channels to promote the investigation. Other contextual factors, which must be considered in evaluating the level of participation, concern the complexity of the company and the numerous stresses and requests on staff at all levels, who carry out additional activities and services daily with an increasingly faster flow. These, which are necessary for the functioning of the services as well as for the continuous improvement process, are added to those of one’s professional routine, to the point of becoming an important part. In any case, since 10% of the population of employees includes over 675 operators, it was considered that this was a good representative number of the population working within the structure. This objective was not achieved. Secondly, the level of participation by profession: not all professional categories are present in a representative manner. Again, the data analysis did not extrapolate the difference in knowledge between healthcare personnel and personnel which is not directly involved in clinical care; this could be the subject of secondary study. A limitation in the analysis of the results also comes from the absence of recent internal comparison data. The last large-scale fact-finding survey carried out by the facility, if one excludes tests belonging to other prevention courses, or field interviews that are regularly carried out during hand hygiene audits, dates back exactly 10 years earlier. The 2013 survey did not include all the questions asked in the current questionnaire. However, the comparison appears eloquent and comforting, with a ‘leap’ from 37.8 to 74.4% in the knowledge level of staff on the topic of hand hygiene.

The regular repetition of these surveys will allow us to have constantly updated information on the current state of affairs, on the monitoring trend, on the evolution of the training program and on any corrections to be made.

Future studies could expand the sample size beyond the 8% participation rate, extending the research across multiple healthcare settings to get a more comprehensive understanding of hand hygiene knowledge and its impact on infection rate. Conducting longitudinal studies would allow researchers to assess whether educational interventions lead to lasting behavioral changes and reduced HAIs over time. Research could also compare different training methods, such as traditional approaches versus innovative techniques like virtual simulations, to determine the most effective one in enhancing hand hygiene knowledge. Investigating behavioral barriers that hinder adherence to hand hygiene guidelines could provide valuable insights into the psychological and social factors that need to be modified. Additionally, specific technologies, such as wearable devices or sensors, could help to improve compliance. Finally, examining how leadership and institutional support influence hand hygiene practices could help to identify the role of mentorship and senior staff in promoting adherence among newbie personnel. Improving these factors, future research can further reduce healthcare-associated infections and enhance patient safety.

## Conclusion

In conclusion, this study underscores the critical importance of healthcare workers’ knowledge and adherence to hand hygiene protocols in reducing healthcare-associated infections. While the overall knowledge level was moderate, significant gaps were identified, particularly in the application of key practices like hand rubbing. The results obtained have made it possible, albeit in a limited way, to understand the extent of the phenomenon to be able to build educational interventions aimed at overcoming those barriers that do not allow adequate adherence to hand hygiene.

The feedback on the results that is intended to be returned not only to all the participants of the survey but to the entire community of employees, becomes fundamental for communicating at what point in the continuum of growth of good practice the staff is, and for its optimization. What appears certain is that the activities related to the measurement and implementation of hand hygiene compliance must be monitored continuously and with methodological rigor, because only by measuring and adapting the objectives of continuous improvement of the quality of care, is there a benefit for patients and the hospitals in which they receive said care.

## Data Availability

The raw data supporting the conclusions of this article will be made available by the authors, without undue reservation.
