# Supplementary material for: Hand hygiene, knowledge and training motivational drives: findings from a survey in a university hospital
Source: Front Public Health. 2024 Dec 18;12:1421324. doi: 10.3389/fpubh.2024.1421324 (PMC11688578; doi:10.3389/fpubh.2024.1421324)
Supplement: Supplementary file 1 [file Data_Sheet_1.pdf]

⏱ **15 minuti**

Indagine sulle conoscenze dell’igiene delle mani negli operatori sanitari della FPG

Gentilissimo,

questa struttura è da tempo coinvolta nel perseguimento della strategia di contrasto alle infezioni correlate all’assistenza sanitaria (ICA) attraverso diverse misure di buona pratica tra cui l'igiene delle mani, straordinario strumento di prevenzione a bassissimo costo, ma di altissima e comprovata efficacia.

L’adesione all’igiene delle mani in FPG, nei 5 Momenti previsti dall’OMS per prevenire le ICA, è, con riferimento al 2022, dell’81,4%. Tale livello di *compliance*, in costante crescita negli ultimi anni e pur decoroso nel risultato, tuttavia deve confrontarsi ancora con il problema delle infezioni, in particolare con quelle sostenute da germi con resistenza multipla agli antibiotici, con il quale la nostra struttura, suo malgrado, convive, e che affronta sistematicamente attraverso un processo coordinato di azioni e di atti agiti dal team Prevenzione e Controllo delle Infezioni (PCI) generato e operante all’interno del percorso JCI.

In aggiunta dunque alle azioni messe continuativamente in campo per il mantenimento del clima organizzativo più favorevole alla *compliance* e all’impegno dei rilevatori, crediamo sia necessario, per misurare il cosiddetto effetto Hawthorne (maggiore adesione da parte degli operatori correlata alla consapevolezza di essere osservati anche se in anonimato), approfondire le conoscenze che guidano i gesti più corretti attraverso un breve questionario che si prega di compilare, in maniera anonima e nel rispetto delle modalità di svolgimento. I risultati guideranno in maniera più mirata le azioni del Team PCI per la sicurezza delle cure e dei nostri pazienti

Con gratitudine, è apprezzata la collaborazione di tutti

F.to Andrea Cambieri   Maurizio Zega   Patrizia Laurenti   Michele Di Donato

* Obbligatoria

Consenso informato

1. Si prega di prendere visione dell'informativa allo studio e al trattamento dei dati al link Documenti:

   Cliccando sul pulsante "Acconsento" dichiara:
   · di aver letto con attenzione le spiegazioni relative allo studio in oggetto;
   · di essere stato informato riguardo alle finalità e agli obiettivi di questo studio;
   · di essere stato informato sull'assenza di rischi correlati allo studio;
   · di aver ricevuto sufficienti garanzie sulla riservatezza delle informazioni ottenute rispondendo all'indagine;
   · di essere consapevole di poter interrompere la compilazione del questionario in qualsiasi momento;
   · di aver liberamente e volontariamente fornito il proprio consenso a partecipare allo studio attraverso la compilazione del questionario anonimo;
   · di essere consapevole che, secondo il rispetto della normativa vigente, i Suoi dati personali saranno usati esclusivamente per scopi di ricerca scientifica;
   · di essere maggiorenne (avere almeno 18 anni);
   · di dare il proprio consenso al trattamento dei dati per finalità relative alla Ricerca;
   · di dare il proprio consenso alla eventuale cessione dei dati in forma anonima a soggetti terzi che utilizzino gli stessi a scopo di studio o ricerca;
   · di essere consapevole che per ogni problema o per eventuali ulteriori informazioni potrà rivolgersi alla UOS Igiene Ospedaliera (mail: servizio.igieneospedaliera@policlinicogemelli.it)

   ◯ Acconsento

   ◯ Non acconsento

Sezione A

2. Caratteristiche sociodemografiche

Genere *

- ◯ Maschio
- ◯ Femmina

3. Età (anni) *

Il numero deve essere compreso tra 18 ~ 100

4. Qualifica professionale *

- ◯ Personale Medico
- ◯ Personale Medico in formazione specialistica
- ◯ Personale Infermieristico
- ◯ Personale Biologo/Farmacista
- ◯ Terapista della riabilitazione
- ◯ OSS
- ◯ Tecnico
- ◯ Ausiliario/OTA
- ◯ Studente
- ◯ Personale amministrativo
- ◯ Altro

5. Corso di laurea *

- Dietistica
- Economia e gestione dei servizi
- Farmacia
- Fisioterapia
- Igiene dentale
- Infermieristica
- Logopedia
- Management dei servizi
- Medicine and surgery
- Medicina e chirurgia
- Odontoiatria e protesi dentaria
- Ortottica ed assistenza oftalmologica
- Ostetricia
- OSS
- Scienze e tecnologie cosmetologiche
- Scienze infermieristiche ed ostetriche
- Scienze riabil. delle profes. sanitarie
- Tecniche audioprotesiche
- Tecniche prev. ambiente e lavoro
- Tecniche di fisiopat. cardiocirc. perf.
- Tecniche di laboratorio biomedico
- Tecniche di radiol. immagini e radio.
- Tecniche ortopediche
- Terapia occupazionale

6. Anno di corso *

Il numero deve essere compreso tra 1 ~ 6

7. Anno di corso *

Il numero deve essere compreso tra 1 ~ 6

8. Specializzazione *

- ◯ Allergologia e immunologia clinica
- ◯ Anatomia patologica
- ◯ Anestesia, rianimaz., terapia intensiva
- ◯ Audiologia e foniatria
- ◯ Cardiochirurgia
- ◯ Chirurgia vascolare
- ◯ Chirurgia generale
- ◯ Chirurgia orale
- ◯ Chirurgia plastica, ricostruttiva ed estetica
- ◯ Chirurgia toracica
- ◯ Dermatologia e venereologia
- ◯ Ematologia
- ◯ Endocrinologia malattie del metabolismo
- ◯ Fisica medica
- ◯ Genetica medica
- ◯ Geriatria
- ◯ Ginecologia ed ostetricia
- ◯ Igiene e medicina preventiva
- ◯ Malattie dell'apparato cardiovascolare
- ◯ Malattie dell'apparato digerente
- ◯ Malattie dell'apparato respiratorio
- ◯ Malattie infettive e tropicali
- ◯ Medicina del lavoro
- ◯ Medicina dello sport e dell'eserc. fisico
- ◯ Medicina d'emergenza-urgenza
- ◯ Medicina fisica e riabilitativa
- ◯ Medicina interna
- ◯ Medicina legale
- ◯ Medicina nucleare
- ◯ Microbiologia e virologia

- ◯ Nefrologia

9. Anno di assunzione (anche non nella stessa UOC o struttura) *

Il numero deve essere compreso tra 1923 ~ 2023

10. Unità operativa attuale *

- ◯ Ortopedia e traumatologia
- ◯ Otorinolaringoiatria
- ◯ Patologia clinica e biochimica clinica
- ◯ Pediatria
- ◯ Psichiatria
- ◯ Radiodiagnostica
- ◯ Radioterapia
- ◯ Reumatologia
- ◯ Scienza dell’Alimentazione
- ◯ Urologia

Sezione B

11. *Hai ricevuto una formazione sull'igiene delle mani negli ultimi tre anni (anche mediante FAD)?* *

- ◯ SI
- ◯ No

12. *Usi abitualmente un prodotto a base alcolica per l'igiene delle mani?* *

- ◯ SI
- ◯ No

13. Quale delle seguenti è la principale via di trasmissione crociata di germi potenzialmente patogeni tra pazienti in una struttura sanitaria? (indica solo una risposta) *

- ◯ Le mani degli operatori sanitari quando non sono pulite
- ◯ Il sistema di areazione della struttura
- ◯ L'esposizione dei pazienti a superfici contaminate (ad es. letti, sedie, tavoli, pavimenti)
- ◯ La condivisione di presidi non invasivi (ad es. stetoscopi, manicotto dello sfigmomanometro, ecc.) tra pazienti

14. Quale delle seguenti azioni di igiene mani previene la trasmissione crociata di germi al paziente? (indica una o più opzioni) *

- ☐ Prima del contatto con il paziente
- ☐ Immediatamente dopo l'esposizione ad un fluido corporeo
- ☐ Dopo il contatto con ciò che sta attorno ad un paziente
- ☐ Immediatamente prima di una procedura pulita/asettica

15. Quale delle seguenti azioni per l'igiene delle mani previene l’infezione dell'operatore sanitario? (indica una o più opzioni) *

- [ ] Dopo un contatto con il paziente
- [ ] Immediatamente dopo l’esposizione ad un fluido corporeo
- [ ] Immediatamente prima di una procedura pulita/asettica
- [ ] Dopo il contatto con ciò che sta attorno ad un paziente

16. Qual è il tempo minimo necessario per un prodotto a base alcolica per eliminare la maggior parte dei germi sulle tue mani? (indica solo una risposta) *

- ○ 20 secondi
- ○ 3 secondi
- ○ 1 minuto
- ○ 10 secondi

17. Dopo il contatto con il paziente, per quali delle seguenti infezioni devi lavare le mani con acqua e sapone e **non** con il prodotto a base alcolica? (indica solo una risposta) *

- ○ Staphylococcus aureus resistente alla meticillina
- ○ Polmonite
- ○ Clostridium difficile
- ○ Epatite A

18. Quali delle seguenti affermazioni sulla frizione delle mani a base alcolica e sul lavaggio delle mani con acqua e sapone sono vere? (indica una o più opzioni) *

- [ ] La frizione con soluzione alcolica è più rapida rispetto al lavaggio delle mani
- [ ] La frizione delle mani provoca secchezza della pelle più del lavaggio delle mani
- [ ] La frizione delle mani è più efficace contro i germi rispetto al lavaggio delle mani
- [ ] Si consiglia di eseguire il lavaggio e la frizione delle mani in sequenza
- [ ] Prima di indossare i guanti è necessaria l’igiene delle mani
- [ ] Se non si tocca il paziente, ma solo le superfici che lo circondano (il suo letto, comodino, flebo, ...), non è importante igienizzare le mani prima di allontanarsi
- [ ] Se si igienizzano correttamente le mani, viene garantita anche l’igiene degli anelli indossati e la sicurezza della mano

Questo contenuto non è stato creato né approvato da Microsoft. I dati che invii verranno recapitati al proprietario del modulo.

Microsoft Forms
